# Spatiotemporal jump detection during continuous film viewing

**DOI:** 10.1167/jov.23.2.13

**Published:** 2023-02-27

**Authors:** Aditya Upadhyayula, John M. Henderson

**Affiliations:** 1 Center for Mind and Brain, University of California – Davis, Davis, CA, USA; 2 Department of Psychology, University of California – Davis, Davis, CA, USA

**Keywords:** predictive processing, visual cognition, spatiotemporal disruptions, saccades, optic flow, OpenCV, FlowNet2, episodic memory, event cognition

## Abstract

Prior research on film viewing has demonstrated that participants frequently fail to notice spatiotemporal disruptions, such as scene edits in the movies. Whether such insensitivity to spatiotemporal disruptions extends beyond scene edits in film viewing is not well understood. Across three experiments, we created spatiotemporal disruptions by presenting participants with minute long movie clips, and occasionally jumping the movie clips ahead or backward in time. Participants were instructed to press a button when they noticed any disruptions while watching the clips. The results from experiments 1 and 2 indicate that participants failed to notice the disruptions in continuity about 10% to 30% of the time depending on the magnitude of the jump. In addition, detection rates were lower by approximately 10% when the videos jumped ahead in time compared to the backward jumps across all jump magnitudes, suggesting a role of knowledge about the future affects jump detection. An additional analysis used optic flow similarity during these disruptions. Our findings suggest that insensitivity to spatiotemporal disruptions during film viewing is influenced by knowledge about future states.

## Introduction

The ability to detect a change in our visual experience plays a major role in our daily life, such as noticing when a car drifts into our lane or noticing and preparing for an exit on the freeway. We also often fail to notice large changes in our visual field owing to the limitations of our visual system. This phenomenon is well documented in the literature as Change Blindness (Henderson & Hollingworth, 2003; Levin, Simons, Angelone, & Chabris, 2002; O'regan, Rensink, & Clark, 1999; Simons & Levin, 1998; Simons & Rensink, 2005). At the core, change blindness happens because of bottlenecks constraining processing at various levels in the visual hierarchy. Some of these bottlenecks are known to us in the form of visual crowding (Freeman & Simoncelli, 2011; Ma, McCloskey, & Flombaum, 2015; Whitney & Levi, 2011), attentional and working memory limitations (Alvarez & Cavanagh, 2004; Luck & Vogel, 1997; Zhang & Luck, 2008), and acuity limitations requiring eye movements (Fehd & Seiffert, 2008; Henderson & Hollingworth, 2003; Smith, Lamont, & Henderson, 2012; Upadhyayula & Flombaum, 2020; Zelinsky, 2001; Zelinsky & Neider, 2008). As a result, we cannot process every bit of information that falls on our retina. Yet, our illusory visual experience tricks us into thinking that we have a uniformly rich, detailed, and spatiotemporally continuous representation of the world. Our visual system overcomes these limitations by making certain assumptions and predictions about the world, which when tampered with could make our vision susceptible and sometimes “blind” to any changes that happen in the real world. Here, we asked how susceptible our visual experience is to the disruptions/changes in the spatiotemporal continuum for dynamic real-world scenes.

A large body of research has investigated change blindness for a variety of natural stimulus types, including text during reading (Angele & Rayner, 2011; Rayner, 2009; Slattery, Angele, & Rayner, 2011; Wilson & Williams, 2018), static pictures of real-world scenes (Hollingworth & Henderson, 2000), magic tricks (Kuhn, Amlani, & Rensink, 2008; Kuhn & Tatler, 2005), and real environments (Levin et al., 2002; Simons & Levin, 1998). However, little research has investigated change blindness for the spatiotemporal properties of dynamic real-world events themselves – a few exceptions include Magliano, Miller, and Zwaan (2001), Magliano and Zacks (2011), and Smith and Henderson (2008). The literature here mostly focused on the consequences of edits while viewing films. Film editing typically creates dramatic spatiotemporal disruptions several times a minute, yet we are often unaware of edits, a phenomenon Smith and Henderson termed “edit blindness.” Edit blindness is influenced by the continuity editing rules in cinematic film that likely help to hide the abrupt edits themselves (Smith & Henderson, 2008).

In the present study, we were interested in whether insensitivity to spatiotemporal disruptions in an unfolding scene is more general than edit blindness during film viewing. In other words, are viewers similarly insensitive to abrupt spatiotemporal disruptions that do not necessarily happen at scene edits, and yet are part of the contrived storytelling of a cinematic film? To our knowledge, this has not been tested before. We therefore investigated this question by asking participants to report any perceived disruptions as they watched continuous video clips with occasional jumps either forward or backward in time as the videos unfolded. A skip-ahead jump – where the film skipped ahead in time – moved the event forward (i.e. further ahead in time than the passage of time warranted), whereas a re-view jump moved the film back to a previous point in time, thus making the participants re-view a portion of the video they had already seen. Analyzing the detections for skip-ahead and re-view jumps should inform us about the underlying systemic constraints of spatiotemporal processing during visual perception.

Like spatial processing, temporal processing in vision has also been shown to be constrained. For example, literature on the attentional blink – a well-known psychological phenomenon – has demonstrated that temporal processing is suppressed immediately following target detection in a rapidly changing stream of letters (Chun & Potter, 1995; Dux & Marois, 2009; Raymond, Shapiro, & Arnell, 1992; Shapiro, Raymond, & Arnell, 1997). Temporal suppression in attentional blink has been shown to be influenced by factors such as difficulty in engaging attention (Kawahara, Enns, & Lollo, 2006; Nieuwenstein, Potter, & Theeuwes, 2009), the psychological refractory period – where current stimulus's processing cost has been shown to mask the subsequent stimulus (Marti, Sigman, & Dehaene, 2012; Welford, 1952), among other factors.

Our visual processing must then overcome the bottlenecks by relying on the knowledge of the world and making suitable inferences about the world. For example, change blindness in natural scenes has been shown to be mediated by the underlying semantics – changes to semantically inconsistent objects with the scene are detected faster than semantically consistent objects (Hollingworth & Henderson, 2000; Rensink, O'regan, & Clark, 1997; Scholl, 2000). Similarly, Smith and Henderson (2008) demonstrated that edit blindness is higher when edits happen during an ongoing action compared to the edits between the scenes. These results demonstrate that scene semantics and event information guide visual processing during spatiotemporal bottlenecks. Additionally, a large body of literature on representational momentum and motion perception also suggests that the visual system anticipates and predicts motion trajectories by projecting forward in time (Freyd & Finke, 1984; Hafri, Boger, & Firestone, 2022; T. Hubbard, 1995; T. L. Hubbard, 1995; T. L. Hubbard, 2005; Kerzel, 2000; Kerzel, Jordan, & Müsseler, 2001). Recent work on event cognition has demonstrated that the boundedness of an event – whether an event is concluded – is rapidly extracted even before the end of the event (Ji & Papafragou, 2022). Participants in this study watched bounded and unbounded video clips with disruptions inserted either at the midpoint or toward the end of the video clip. They found that the type of the event (bounded/unbounded) interacted significantly with whether participants were able to detect disruptions at these locations – suggesting that boundedness of an event is anticipated before the event completion. Finally, prediction of unfolding information has also been shown to influence other cognitive domains, such as mechanisms of reading and language comprehension (Ferreira, Foucart, & Engelhardt, 2013; Hale, 2001; Levy, 2008; Rayner, 2009). Collectively, these findings suggest visual processing benefits from the top-down knowledge during bottlenecks.

Moreover, relying on the knowledge of the external world could also increase efficiency of our visual processing by avoiding processing wherever unnecessary. We therefore hypothesized that if our visual processing benefits from knowledge of the unfolding information of the world, then participants’ reports of spatiotemporal disruptions should reflect this knowledge. Specifically, a disruption wherein the input matches the assumptions made about the external world would be less likely be detected compared to a disruption violating such assumptions. In such a scenario, the disruption is less likely to be noticed when less additional processing is needed to bridge the disruption as a result of the match between the input and knowledge of the external world. In the experiments to follow, we test this hypothesis using both eye tracking and behavioral measures and discuss potential underlying mechanisms that could facilitate detecting spatiotemporal jumps during film viewing.

## Experiment 1

In the first experiment, we investigated how sensitive participants are to spatiotemporal disruptions in video clips. The video clips from a film without edits were used so that any lack of sensitivity could not be due to edit blindness. We used a saccade contingent display change paradigm to mask the spatiotemporal discontinuities themselves that are induced by a video jump. Saccadic suppression is a widely reported phenomenon in the eye tracking literature where information from the external world is suppressed during a saccadic event (Breitmeyer & Ganz, 1976; Matin, 1974). The visual processing must overcome this bottleneck by relying on the knowledge of the external world to provide a rich and spatiotemporally continuous visual experience. We therefore hypothesized that any saccade contingent display changes that are in line with our expectations of how information unfolds may be detected less frequently because they are not violating the expectations made by the visual system during information absent episodes. On the other hand, any changes violating expectation would be detected more frequently. In terms of the spatiotemporal jumps, we expect skip-ahead jumps to be detected less frequently compared to the re-view jumps.

### Methods

#### Participants

Thirty-two undergraduate students from University of California – Davis participated in this study in exchange for course credits. We sought to test between 30 and 35 participants based on typical sample sizes for eye tracking studies involving film viewing. Two participants were excluded from the study due to noisy data, and errors in eye tracking calibration, thus leaving 30 participants for further analysis. All participants had normal to corrected normal vision by self-report. The protocols of all the reported experiments were approved by the Institutional Review Board (IRB) of the University of California – Davis. In accordance with the approved IRB, demographic information was collected anonymously and without identifiers linked to specific experimental results, used only for year-end reporting.

#### Stimuli and apparatus

Video clips without audio from the movie *1917* were used in this study. This movie was chosen because it does not have any perceivable scene edits. Thus, any interference of the scene edits in detecting video jumps were avoided. A total of 37 video clips were extracted from the first half of the movie. Each clip was approximately 1 minute long. All the video clips contained some degree of camera panning/zooming. The generated video clips were re-encoded using *ffmpeg* to separate audio and video streams from the movie clips, and the audio stream was discarded. The final re-encoded video clips were of the dimensions 1920 × 1080 pixels (high definition [HD] format), with a bit rate of 24 frames per second. Two additional versions of each video clip were then generated with the video clips starting either 500 msec or 1000 msec later than the start of the original clip. This way, each video clip had two delayed versions of the same clip.

Stimuli were presented at a screen resolution of 1920 × 1080 using a 27 inch LED gaming monitor with 60 Hz refresh rate. The experiment was created and presented using Experiment Builder (version 2.3.38), an SR Research software program. We used an Eyelink 1000 plus tower mount for this study. Eye tracking data was sampled at 1000 Hz, and participants’ right eyes were tracked with default saccade and blink settings. Participants viewed the stimuli from 80 cm away from the screen.

#### Design and procedure

**Figure 1.**
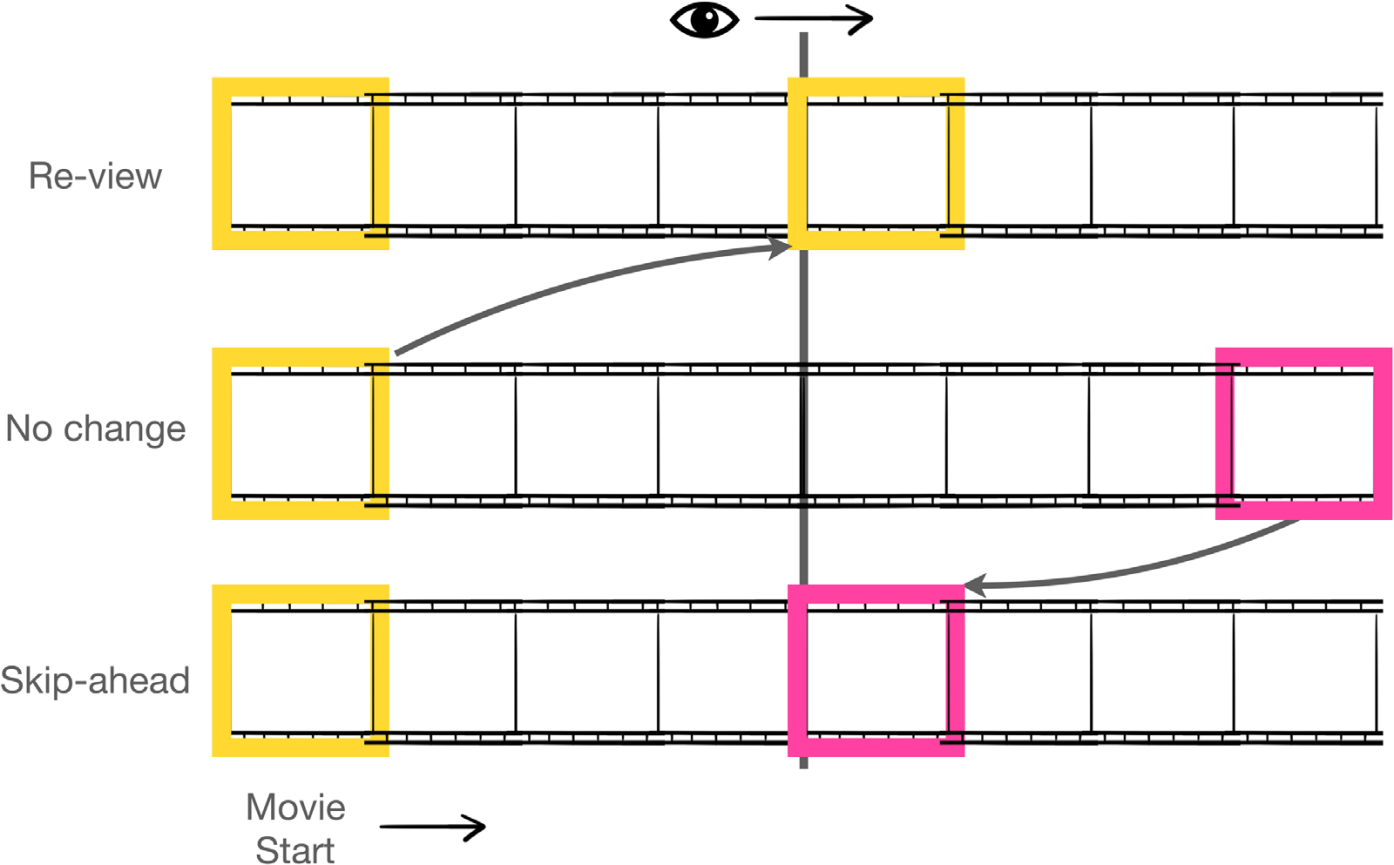
Schematic of the saccade contingent temporal disruption paradigm. During occasional saccades, the video clips either skipped-ahead in time (highlighted in pink) or were re-viewed (highlighted in yellow).

See Figure 1 for a schematic of the experiment. Each trial began with a nine-point calibration routine that mapped eye position to screen coordinates. Calibration was accepted when the average error was less than 0.49 degrees and the maximum error was less than 0.99 degrees. Participants were recalibrated as needed throughout the study. Participants completed two practice trials followed by 35 test trials. Trials began with participants fixating on a central point on the screen. Participants pressed a button to begin the trial. This fixation also served as a “drift check” for the eye tracker to record any shift in gaze position since calibration. The fixation point was then replaced by the video clip. Only one video was visible to the participant at any one time, but all three versions of the video (unaltered, first 500 ms later, and the first 1000 ms later) began playing simultaneously at the start of the trial. Every 4000 ms to 6000 ms, the program activated a velocity trigger which checked for a velocity of at least 140 degrees/sec. Upon reaching the velocity threshold, the display was changed on the screen. This velocity cannot be attained during smooth pursuit and so indicates a saccade (Takahashi, Uemura, & Fujishiro, 1983). Display changes were made during saccades when the visual system suppresses visual transients (Matin, 1974). The high velocity criterion also ensured a longer saccade, allowing enough time to change the video during the saccade and before the next fixation began. Once the velocity trigger was reached, the program switched the visible video with one of the other concurrently playing versions of the video. This produced a seamless video on the monitor that occasionally (during a saccade) either skipped ahead or backward in time by 500 or 1000 ms.

Participants were instructed to respond with a button press if they detected a change. Responses were counted as correct if the participant pressed the button within 2000 ms after the change was initiated. Responses beyond this cutoff were excluded from all analyses. Once the visible video changed, another 4000 ms to 6000 ms delay occurred before the velocity trigger was activated again. Each change was randomly selected with the constraint that changes always occurred in pairs: a change in one direction was always followed by a return change in the opposite direction and of an equal magnitude. This was done to balance the number of skip-ahead and re-view changes per trial. Each trial contained five changes: one re-view 1000 ms (where participants had to re-watch the last second in the clip), one re-view of 500 ms, one skip-ahead of 1000 ms (where participants skipped ahead into the video by 1 second), and one skip-ahead 500 ms. In addition, a 0 ms change was also used during which the program did not update the visible video but went through the velocity detection algorithm and reloaded the same video. This 0 ms control condition provided a baseline for determining the change detection false alarm rate. The trial ended once the participant made enough (5 in this case) critical saccades to change the display.

### Data analysis and results

The materials necessary for the data analysis are available via the OSF repository (https://osf.io/j95z3/). We analyzed participant keypresses in the primary analysis. Participant keypresses were coded as 1 if they pressed the button within 2 seconds of the display change, or 0 if they missed it. To make sure that the display changes were reliable, the following instances were excluded from our analysis. (1) Instances where the display failed to change during the saccade of interest. On average, the display changed 8.5 ms after the initiation of the critical saccade, well within the typical saccade duration of 30 to 40 ms (Findlay, & Gilchrist, 2003; Rayner, 2009). (2) If the change was triggered by a blink or by noise in the eye-tracking data instead of a saccade. (3) If the saccades were greater than 70 msec. These instances could be tracking data losses mislabeled as saccades. We therefore wanted to avoid any changes that were triggered by mislabeled saccades. (4) Duplicate keypresses of the same change (i.e. if the participants pressed a button twice within the specified 2 second limit). Given the categorical nature of the dependent variable, we applied generalized linear mixed effects (GLME) models using the R “lme4” package version 1.1–28; (Bates, Kliegl, Vasishth, & Baayen, 2015) in the R programming environment (version 4.1.1, R Core team, 2019). The binary dependent variable was jump detection. Change direction, change magnitude, and saccade duration were the predictor variables. Trials and participants were treated as random effects with direction, magnitude, and saccade duration as the random slopes – resulting in a maximal model containing all fixed effects predictors as random slopes. Additionally, we ran a minimal variant of the model – with trial and participants as random effects at the intercept level. A model comparison analysis did not reveal a statistically significant difference between the maximal and the minimal models (χ^2^ = 21.449, *P* = 0.862). Furthermore, the minimal model had a lower Akaike information criterion (AIC) score compared to the maximal model. Comparing models with best fit and retaining them for analyses has also been discussed elsewhere in the literature (Bates et al., 2015; also see Barr, Levy, Scheepers, & Tily, 2013 for an alternative approach to model fitting). Therefore, the minimal model was used for the remainder of analysis. Accordingly, any biases/errors at the participant and trial level were accounted for as random effects at the intercept level thus discounting the effects of random slopes.

**Figure 2.**
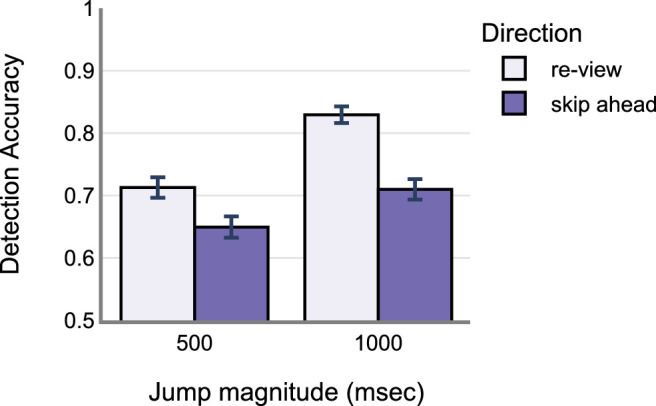
Observed behavior data from 29 participants in Experiment 1. Error bars indicate standard error of mean (SEM).

**Figure 3.**
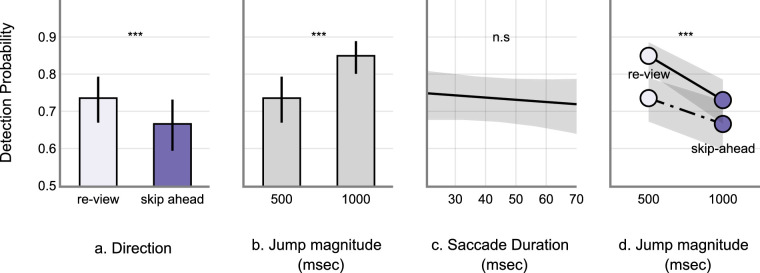
GLME model predictions. (**a**) Marginal estimate of jump direction. (**b**) Marginal estimate of jump magnitude. (**c**) marginal estimate of saccade duration in change detection. (**d**) Interaction plot. Error bars and the shaded regions represent 95% CI. CI, confidence interval.

**Figure 4.**
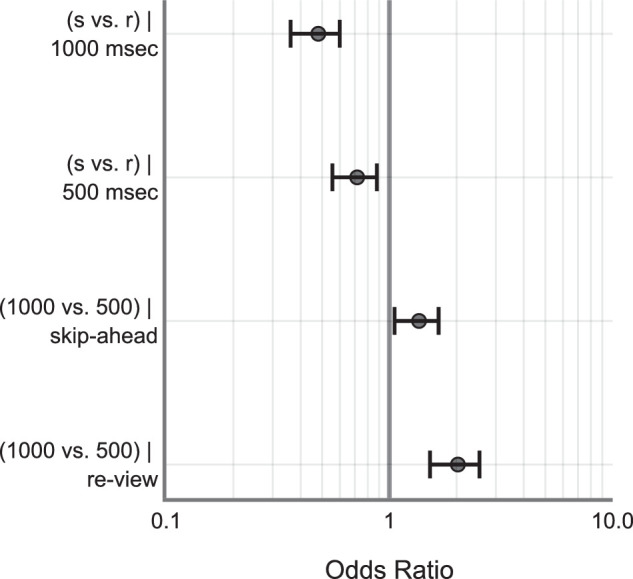
Tukey post hoc comparisons. Higher odds ratio indicates a better chance of detecting jumps, and vice-versa. Error bars indicate 95% CI of the estimates. CI, confidence interval; s, skip-ahead; r, review.

**Figure A1.**
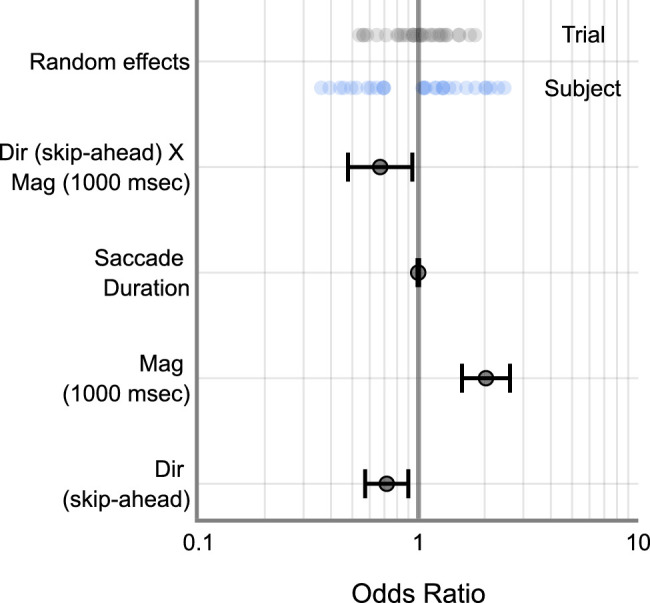
Estimates of the GLM model. Error bars indicate the 95% CI. Each estimate provides the odds for a unit increase in the predictor variable compared to the intercept (Re-view condition's 500 msec). Light gray dots indicate random effects from trial level. Blue dots indicate the random effects from subjects.

Marginally, skip-ahead jumps were less frequently detected compared to the re-view jumps (skip-ahead: M = 0.67, SD = 0.46 and re-view: M = 0.77, SD = 0.41). Further, 500 ms jumps were also less frequently detected compared to the 1000 ms jumps (500 ms: M = 0.68, SD = 0.46 and 1000 ms: M = 0.77, SD = 0.42). The raw data is shown in Figure 2. The same is also true for the model fits. See Appendix (Table A1 and Figure A1) for model estimates and estimate plot. Drop1 model comparisons – where predictor variables were removed one at a time systematically to account for the main effects – revealed a significant main effect of change direction (χ^2^ = 36.41, *P* < 0.001), significant main effect of change magnitude (χ^2^ = 32.5, *P* < 0.001), and a significant interaction between change direction and magnitude (χ^2^ = 5.423, *P* < 0.05). However, there was no significant main effect of saccade duration on detection accuracy (χ^2^ = 0.58, *P* = 0.447) – see Figure 3. Post hoc tests revealed that skip-ahead jumps were detected less frequently in both magnitude conditions compared to the re-view jumps. Similarly, 500 ms jumps were less frequently detected compared to 1000 ms in both the direction conditions – see Table 1 and Figure 4. Finally, analysis of the false alarm data – for example, 0 msec display change after the critical saccade – revealed that participants only reported false alarms 0.2% of the time in all such changes, which is much lower than the detection rate when the display changed.

**Table 1.** Post hoc comparisons within each predictor variable while controlling for the interaction effects from other predictor variables.

Direction	Magnitude	Contrast	Odds ratio	SE	df	Null	z-ratio	*P* value
R	–	1000/500	2.028	0.260	Inf	1	5.519	**<0.001**
S	–	1000/500	1.358	0.156	Inf	1	2.663	**0.028**
	500	(S)/(R)	0.717	0.083	Inf	1	−2.875	**0.015**
	1000	(S)/(R)	0.480	0.061	Inf	1	−5.757	**<0.001**

R, re-view; S, skip-ahead.

### Discussion

This experiment investigated how sensitive our visual system is to spatiotemporal disruptions. We used a saccade contingent display change paradigm during film viewing, where a video clip skipped ahead in time, or skipped to an earlier point in time. We hypothesized that our visual system would be relatively insensitive to spatiotemporal disruptions. In addition, we hypothesized that knowledge about unfolding information would affect sensitivity to spatiotemporal disruptions, thus leading to a significant difference in reports between skip-ahead and review reports made by the participants. Overall, we found that jumps were missed approximately 28% of the time. Participants reported the re-view jumps more frequently (approximately 10% higher) than the skip-ahead jumps. Larger magnitude jumps (1000 ms) were detected more frequently (approximately 10% higher) compared to the 500 ms jumps. Finally, there was no effect of saccade duration on the detection accuracy of the video jumps.

Overall, the results suggest that detection of spatiotemporal disruptions is influenced by the direction and magnitude of the spatiotemporal change. Specifically, a change to a later point in time (in the direction of unfolding information) is noticed by the participants less frequently than a change to an earlier point in time, considering that the videos jumped as much as 1000 ms in time in both the directions. To investigate whether viewers’ insensitivity is based on the degree of spatiotemporal disruption, Experiment 2 further increased the magnitude of jumps to 2000 ms.

## Experiment 2

This experiment is a conceptual replication of Experiment 1 with a minor modification. Specifically, we tested viewers’ insensitivity to larger magnitude jumps of 1000 and 2000 ms as they watched the videos unfold in time.

### Participants

Thirty-three undergraduate students from University of California – Davis, participated in this study in exchange for course credits. Four participants were excluded from the study due to noisy data and errors in eye tracking calibration, leaving 29 participants for further analysis. All participants had normal to corrected normal vision by self-report. The protocols of all the reported experiments were approved by the IRB of the University of California – Davis. In accordance with the approved IRB, demographic information was collected anonymously and without identifiers linked to specific experimental results, used only for year-end reporting.

### Stimuli and apparatus

The stimuli were the same as in the Experiment 1 except with one modification. Two additional versions of each video clip were generated with the video clips starting either 1000 ms or 2000 ms later than the start of the original clip.

### Design and procedure

The design and procedure for Experiment 2 were the same as in the Experiment 1.

### Data analysis and results

Analysis protocols and the data exclusion criteria for this experiment were the same as that of Experiment 1. The materials necessary for the data analysis are available via the OSF repository (https://osf.io/j95z3/). On average, the display in this experiment changed 8.6 ms after a saccade was detected by the eye tracker – like Experiment 1. However, about 54% of the data was excluded from our analysis in this experiment compared to the 28% in Experiment 1. Most of the display changes in the excluded data were triggered by blinks mislabeled as saccades that had duration greater than 70 msec – for example, about 37% of the display changes that met the pre-processing criteria from Experiment 1 had saccade durations greater than 70 msec. The remainder of the data was analyzed using GLME models. Similar to Experiment 1, a model comparison analysis between the maximal and the minimal models (i.e. with and without the random slopes) did not reveal a statistically significant difference (χ^2^ = 29.617, *P* = 0.381). Accordingly, participants and trials were accounted for as random effects at the intercept level thus discounting the effects of random slopes.

**Figure 5.**
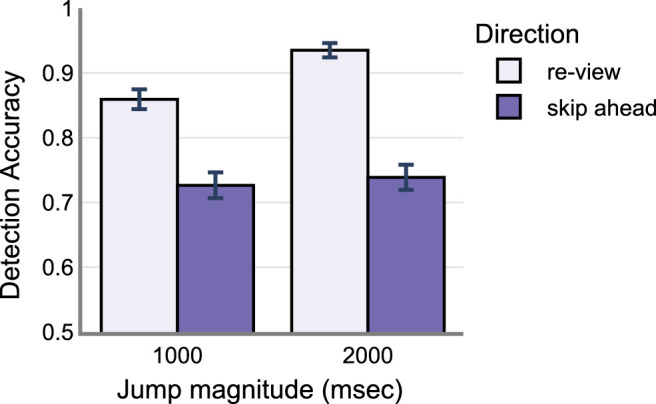
Observed behavior data from 30 participants in Experiment 2. Error bars indicate standard error of mean (SEM).

**Figure 6.**
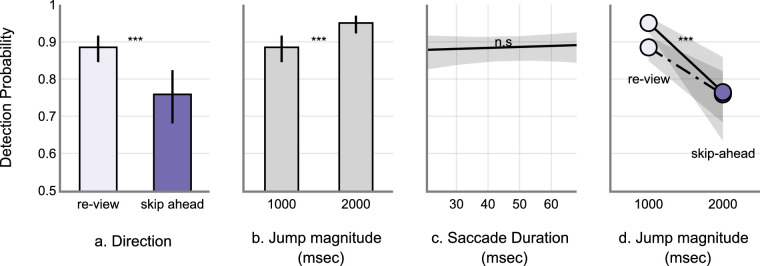
GLME model predictions. (**a**) Marginal estimate of jump direction. (**b**) Marginal estimate of jump magnitude. (**c**) marginal estimate of saccade duration in change detection. (**d**) Interaction plot. Error bars and the shaded regions represent 95% CI. CI, confidence interval.

**Figure 7.**
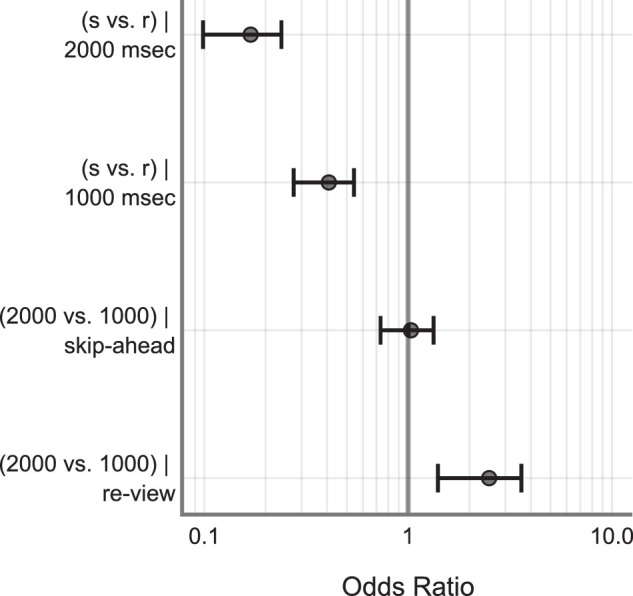
Tukey post hoc comparisons. Higher odds ratio indicates a better chance of detecting jumps, and vice-versa. Error bars indicate 95% CI of the estimates. CI, confidence interval; s, skip-ahead; r, review.

**Figure A2.**
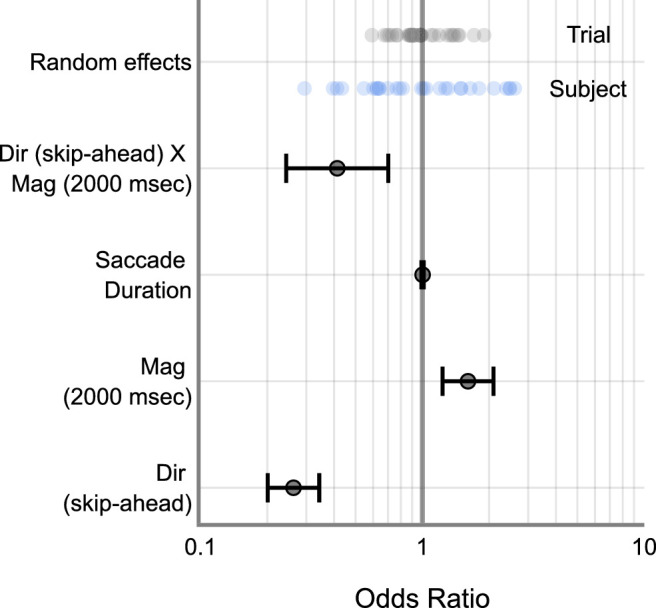
Estimates of the GLM model. Error bars indicate the 95% confidence interval (CI). Each estimate provides the odds for a unit increase in the predictor variable compared to the intercept (Re-view condition's 1000 msec). Light gray dots indicate random effects from trial level. Blue dots indicate the random effects from subjects.

On average, skip-ahead jumps were detected less frequently compared to the re-view jumps (skip-ahead: M = 0.73, SD = 0.44 and re-view: M = 0.89, SD = 0.30). The 1000 ms jumps were detected less frequently compared to the 2000 ms jumps (1000 ms: M = 0.79, SD = 0.40 and 2000 ms: M = 0.83, SD = 0.37). Raw data is shown in Figure 5. See the Appendix (Table A2 and Figure A2) for model estimates and estimate plot. Drop1 model comparison analyses revealed a significant main effect of change Direction (χ^2^ = 101.66, *P* < 0.001), significant main effect of change magnitude (χ^2^ = 6.51, *P* = 0.01), and a significant interaction between change direction and magnitude (χ^2^ = 10.84, *P* < 0.001). There was no significant main effect of saccade duration on detection accuracy (χ^2^ = 0.17, *P* = 0.67) – see Figure 6. Post hoc analyses revealed that skip-ahead jumps were detected less frequently in both magnitude conditions compared to the re-view jumps. The 1000 msec jumps were detected less frequently compared to the 2000 msec jumps in the re-view condition. However, there was no significant difference between 1000 and 2000 msec jumps in the skip-ahead condition – see Table 2 and Figure 7. Finally, false alarm rates were about 0.3 %, similar to that found in Experiment 1.

**Table 2.** Post hoc comparisons within each predictor variable while controlling for the interaction effects from other predictor variables.

Direction	Magnitude	Contrast	Odds ratio	SE	df	Null	z-ratio	*P* value
R	–	2000/1000	2.498	0.562	Inf	1	4.066	**<0.001**
S	–	2000/1000	1.033	0.154	Inf	1	0.215	0.995
	1000	(S)/(R)	0.408	0.069	Inf	1	−5.330	**<0.001**
	2000	(S)/(R)	0.169	0.036	Inf	1	−8.343	**<0.001**

R, re-view; S, skip-ahead.

### Discussion

This experiment was a conceptual replication of Experiment 1. Here, we investigated whether participants are still sensitive to spatiotemporal disruptions even at larger magnitude. We increased the magnitude of spatiotemporal jumps to a 2000 msec maximum from 1000 msec in Experiment 1. Overall, we found that jumps were missed approximately 19% of the time. Jumps in the skip-ahead direction were detected less frequently compared to the re-view direction replicating the findings of Experiment 1. Similarly, 1000 msec jumps were detected less frequently compared to 2000 msec. Post hoc analyses further revealed that skip-ahead jumps were not significantly different between 1000 and 2000 msec (1000 msec: M = 0.726, SD = 0.44 and 2000 msec: M = 0.738, SD = 0.43) – see Figure 6. This contrasts with the findings of Experiment 1 where skip-ahead jumps were detected more for 1000 msec compared to skip-ahead in the 500 msec – see Figure 4. It is possible that detection accuracy saturated for jumps greater than 1000 msec – for example, jumps greater than 1000 msec are likely detected with similar accuracy. This is also supported by a similar detection rate for 1000 msec skip-ahead jumps in Experiment 1 data (M = 0.70, SD = 0.45). The re-view condition however showed a significant difference between 1000 and 2000 msec jumps – see Figure 7. A possible explanation for this difference concerns memory – where having rewatched portions of the videos in the re-view condition could have aided in better detection. We discuss this more in detail in the General Discussion section. Finally, like in Experiment 1, saccade duration was not a significant predictor of detection accuracy. Overall, these results replicate the basic findings of Experiment 1 in demonstrating that skip-ahead jumps are detected less frequently (approximately 13% lower) compared to the re-view jumps. Furthermore, they also demonstrate that this pattern holds for larger magnitude jumps (i.e. jumps as big as 2000 ms were often missed by participants).

In order to understand why the skip-ahead jumps are detected less frequently compared to the re-view jumps, we next investigated whether the camera attributes of the video systematically differed for skip-ahead and re-view jumps. Videos unfolding over time have a particular direction in which the information flows – the optic flow. Optic flow for skip-ahead jumps could be different compared to re-view, and therefore contribute toward the observed differences between skip-ahead and re-view detections. We therefore investigated the role of optic flow in detection of spatiotemporal disruptions in video clips in a subsequent analysis.

### Analysis of the optic flow patterns during change detection

To study if the optic flow patterns could predict change detection performance in this paradigm, we analyzed the combined data from both Experiments 1 and 2. Optic flow is the pattern of motion between objects, surfaces, and edges in a visual scene caused by the relative motion between the observer and the scene. It describes the flow of the content from one frame to another in a given sequence of scenes. Our visual system relies on optic flow for various functions, such as perception of self-motion (Gibson, 1947; Warren & Hannon, 1988), visual guidance for action planning (Warren, 2021), balance and posture control (Stoffregen, 1985). See (Niehorster, 2021) for a review on optic-flow. The observed motion flow is often highly correlated in time because information changes gradually across the samples that make up our visual experience. It is therefore possible that the optic flow in the skip-ahead condition might be highly correlated with the flow prior to the jump, compared to the re-view condition. As a result, when a video skips ahead in time, an optic flow-based mechanism of jump detection might be less likely to notice a skip ahead jump because of the strong resemblance in optic flow patterns before and after the jump. On the other hand, re-view jumps happen in the opposite direction (i.e. away from the direction of the motion flow). The optic flow patterns before and after the jump in this case could bear a weaker resemblance, and therefore could aid in increased detection rates in the re-view condition. We hypothesized that optic flow similarity – for example, similarity between the flow patterns before and after the jump – is significantly different for skip-ahead and re-view conditions. Furthermore, if detecting spatiotemporal jumps relies on optic flow similarity, we should expect a decline in detection rates as the optic flow similarity increases.

#### Materials

We used the data from 59 participants combined from Experiments 1 and 2. For each participant and each video clip they saw, we extracted a set of three frames – two before and one immediately after the critical time points where the videos jumped in time. The extracted frames were then scaled down by a factor of 0.25 to facilitate computation time, thus scaled down from 1920 × 784 resolution to 480 × 196 pixels per image. We used OpenCV, a python computer vision library to calculate the optic flow between a given two pairs of frames in the sequence. Thus, the three extracted frames resulted in two optic flow patterns – one before the jump, and the other immediately following the jump. The optic flow patterns were computed for both the skip-ahead and re-view conditions across all the three (500, 1000, and 2000 ms) magnitude jumps combined from both the experiments.

### Data analysis and results

The materials necessary for the data analysis are available via the OSF repository (https://osf.io/j95z3/). The computed optic flow arrays (size = 480 × 196 × 2) contained both magnitude and angle information pertaining to the optic flow for every pixel in the image. Each of the optic flow arrays before and after the jump was vectorized to compute the correlation between them. A similarity index of 1 would imply that the optic flow before and after the jump are identical to each other, and a similarity index of -1 would imply a strong dissimilarity. The optic flow similarity index was computed for each of the re-view, skip-ahead jumps across 500, 1000, and 2000 ms magnitudes for each participant and the video clip. The computed optic flow similarity index was then paired against the behavior data included in the analyses of Experiments 1 and 2. Thus every detected/missed jump from the participants had a corresponding optic flow similarity index pertaining to the change. Furthermore, outliers above 95th percentile in the optic flow similarity distribution were excluded from the analyses to preserve the homogeneity in the data – thus leading to an additional 10% data exclusion from the two experiments combined.

**Figure 8.**
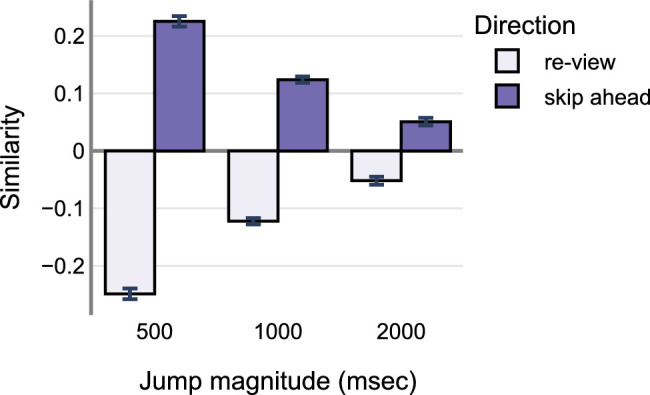
Optic flow similarity as a function of jump direction and magnitude. The similarity index is computed by correlating optic flow arrays before and after the critical points where the videos jumped ahead in time.

On average, the optic flow similarity index was higher for skip-ahead jumps compared to the re-view jumps (skip-ahead: M = 0.14, SD = 0.26 and re-view: M = −0.14, SD = 0.26). Similarity index averaged across jump magnitudes was similar (500 ms: M = −0.004, SD = 0.37; 1000 ms: M = 0.002, SD = 0.28; and 2000 ms: M = 0.005, SD = 0.22). The raw data are shown in Figure 8. A two way ANOVA with direction and magnitude as the predictor variables and the similarity index as the dependent variable revealed significant main effects of direction (F(1, 5101) = 1596.46.190, *P* < 0.001). There was no main effect of magnitude: F(2, 5101) = 0.86, *P* = 0.423). The interaction between direction and magnitude was significant (F(2, 5101) = 165.62, *P* < 0.001). Tukey's post hoc comparisons revealed a significant pairwise comparisons across all the levels of direction and magnitude conditions (all *P* values < 0.001). These results demonstrate that optical flow similarity is significantly predicted by both the direction and magnitude of the jumps, and the interactions between them.

**Figure 9.**
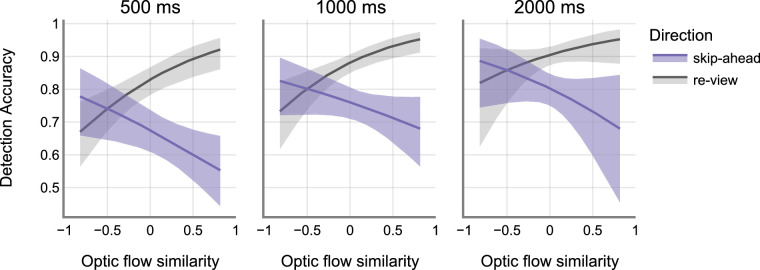
Detection accuracy as a function of optic flow similarity. Shaded regions indicate the 95% CI of the estimates. CI, confidence interval.

**Figure A3.**
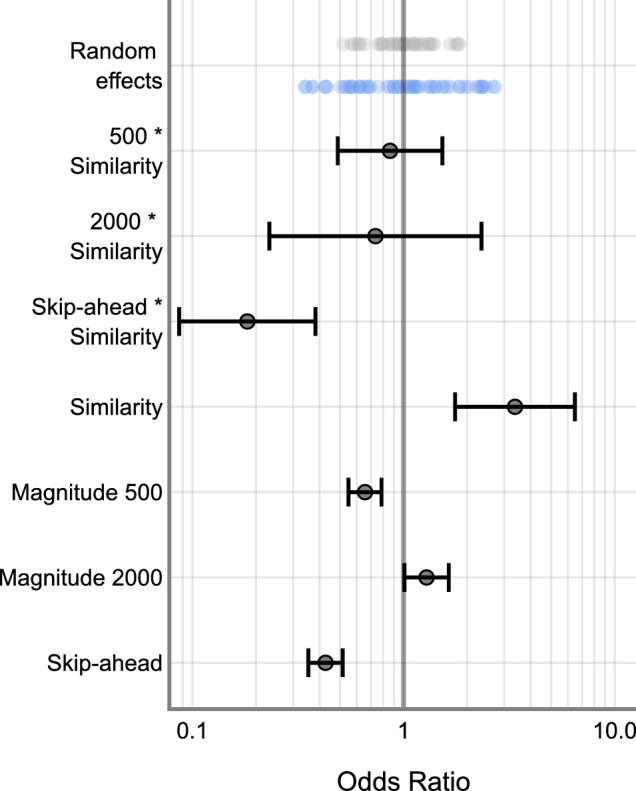
Estimates of the GLM model. Error bars indicate the 95% confidence interval (CI). Each estimate provides the odds for a unit increase in the predictor variable compared to the intercept (re-view condition's 1000 msec, and optic flow similarity 0). Light gray dots indicate random effects from trial level. Blue dots indicate the random effects from subjects.

To check if optic flow similarity index predicts detection rates, we applied GLME models using the R “lme4” package (version 1.1–28; Bates et al., 2015) in the R programming environment (version 4.1.1, R Core team, 2019). The binary variable Detect was the dependent variable, and optic flow similarity index, and change direction were the predictor variables. Similar to the previous experiments, a model comparison analysis between the maximal and the minimal models (i.e. with and without the random slopes) did not reveal a statistically significant difference (χ^2^ = 69.046, *P* = 0.509). Accordingly, participants and trials were accounted for as random effects at the intercept level thus discounting the effects of random slopes. See the Appendix (Table A3 and Figure A3) for model estimates and estimate plot. Drop1 predictor model comparisons revealed significant main effect of direction (χ^2^ = 82.01, *P* < 0.001), and a significant main effect of magnitude (χ^2^ = 25.452, *P* < 0.001). On average, detection accuracy was lower for skip-ahead jumps compared to the re-view jumps (re-view: M = 0.87, SEM = 0.13 and skip-ahead: M = 0.75, SEM = 0.12). Detection accuracy also increased as a function of jump magnitude (500 ms: M = 0.81, SEM = 0.14; 1000 ms: M = 0.87, SEM = 0.13; and 2000 ms: M = 0.90, SEM = 0.15) – replicating the findings from Experiments 1 and 2. There was no significant main effect of optic flow similarity (χ^2^ = 0). However, the interaction between direction and optic flow similarity was significant (χ^2^ = 19.9, *P* < 0.001). On average, detection accuracy for skip-ahead condition decreased with increased optic flow similarity (500 ms: slope = −0.636, SE = 0.272; 1000 ms: slope = −0.489, SE = 0.305; and 2000 msec: slope = −0.796, SE = 0.568). We observed the opposite trend for the re-view condition – where detection accuracy increased with increased optic flow similarity (500 ms: slope = 1.067, SE = 0.286; 1000 ms: slope = 1.215, SE = 0.333; and 2000 ms: slope = 0.908, SE = 0.599) - see Figure 9. The interaction between magnitude and optic flow similarity was not significant (χ^2^ = 0.4, *P* = 0.818). We therefore conducted post hoc analyses ignoring the magnitude variable – thus collapsing the data into skip-ahead and re-view conditions. Tukey's post hoc analysis comparing the skip-ahead and re-view conditions showed a significant difference in the trends between the two conditions (re-view – skip-ahead: estimate = 1.7, SE = 0.379, z = 4.491, *P* < 0.001). These results show that optic flow similarity affects detection rate differently for re-view versus skip-ahead conditions.

### Discussion

This study investigated the role of optic flow in detecting spatiotemporal disruptions while watching the movie *1917*. We hypothesized that skip-ahead jumps would be experienced differently in terms of the optic flow similarity compared to the re-view jumps. Accordingly, the results show that optic flow similarity for the frames before and after the jump is higher for skip-ahead compared to the review condition - see Figure 8. Frames before and after the skip-ahead jump were more “similar” to each other in terms of optic flow. In addition, the optic flow similarity decreased as the jump magnitude increased (i.e. flow similarity in 2000 ms jumps was much lower compared to the flow similarity at 500 and 1000 ms). On the other hand, frames before and after the re-view jump were more dissimilar to each other in terms of the optic flow. Furthermore, this dissimilarity decreased as the jump magnitude decreased (i.e. flow patterns before and after the jump were highly dissimilar to each other at 500 ms compared to 1000 and 2000 ms – see Figure 8). A potential explanation for the optic flow dissimilarity in the re-view condition concerns the underlying scene content and the film editing techniques. The optic flow resulting from an ongoing action, such as throwing a ball, would be more similar to the future information given the non-symmetric nature of the action. Similarly, camera movements such as zoom would also result in asymmetric trajectories from start to end of the movement and vice-versa. Together, these could explain why the optic flow patterns were more dissimilar in the re-view condition compared to the skip-ahead. These results demonstrate unique optic flow similarity signatures for re-view versus skip-ahead jumps.

We then investigated if optic flow similarity could predict jump detections. Specifically, we hypothesized that more similar optic flow patterns before and after the jump would likely result in lower jump detection rates. Our results demonstrate that optic flow similarities affect detection rates differently in skip-ahead versus re-view conditions – see Figure 9. Specifically, we observed that high similarity between optic flow patterns resulted in lower detection rates in the skip-ahead condition – confirming our hypothesis. However, the opposite was true for the re-view condition where the detection rates increased with increased optic flow similarity. This was true across all the jump magnitudes – see Figure 8. We discuss the implications of these results in the General Discussion below.

### General discussion

This study investigated whether participants are sensitive to spatiotemporal visual disruptions while watching film videos. Previous work on edit blindness (Smith & Henderson, 2008) has demonstrated that participants often miss scene edits that adhere to continuity rules of the film narrative while watching movies. Failure to notice scene edits reflects the presence of a higher-level mechanism that fills in missing information by making suitable predictions as the world unfolds (Magliano et al., 2001; Magliano & Zacks, 2011; Smith & Henderson, 2008). Whether such prediction-based mechanisms generalize to film viewing beyond scene edits is not clearly understood. For example, are viewers similarly insensitive to abrupt spatiotemporal disruptions that do not necessarily happen at scene edits, and yet are part of the contrived storytelling of a cinematic film? To our knowledge, there is very little research investigating how spatiotemporal disruptions are perceived in film viewing.

We therefore explored this question as participants watched movie clips without any audio and scene edits, by making the clips occasionally skip ahead in time or to a past point in time, thus disrupting the spatiotemporal continuum. These jumps only happened during the saccades made by participants – thus rendering participants functionally blind to the changes. On average, participants detected skip-ahead jumps (65 and 75% respectively in Experiments 1 and 2) less frequently compared to the re-view jumps (75 and 90%, respectively, in Experiments 1 and 2) – see Figure 3a and Figure 5a. The results also showed that larger magnitude jumps were easier to detect compared to the smaller magnitude jumps – see Figure 3b and Figure 5b. Across the two experiments, the measured false alarm rates were very low (about 0.2% in Experiment 1, and 0.3% in Experiment 2). Collectively, the results from Experiments 1 and 2 demonstrate that participants failed to detect the jumps about 10% to 30% of the time.

It should be noted that participants were instructed beforehand to look for any jumps in the video. Participants in the original change blindness study (Simons & Levin, 1998) were not aware of the change until the stimulus had been presented. They subsequently missed any changes in the stimuli about 65% of the time. The miss rate further dropped to <5% when they were instructed to look for changes beforehand. Therefore, failing to detect the changes between 10% and 30% despite prior instructions further highlights the limitations of our visual processing.

Our data also demonstrate that jump detections were modulated by the optic flow similarity between the flow vectors before and after the jumps. Furthermore, the magnitude of the jumps also played a significant role in detection. These results implicate the role of stimulus content in detecting spatiotemporal continuity. Even though the jumps happened during saccades which typically last anywhere between 20 and 40 ms (Findlay et al., 2003), stimulus properties did influence jump detections. Participants detection rates varied as a function of jump direction, magnitude, and optic flow similarity. This suggests that the jump blindness is unlikely due to the physiological constraints of our visual processing such as the psychological refractory period (Marti et al., 2012; Welford, 1952), or difficulty engaging in attention (Kawahara et al., 2006; Nieuwenstein et al., 2009). Rather, they highlight the limitations specific to the underlying mechanisms of our visual processing. We review some potential mechanisms below.

#### Why were skip-ahead jumps detected less frequently compared to re-view jumps?

Our data across two experiments consistently demonstrated that skip-ahead jumps were detected less frequently compared to the re-view jumps across all the magnitude conditions. An intuitive explanation for this difference is that re-views are simply easier to detect because participants can remember already having watched portions of the clips they are now rewatching. Indeed, memory driven effects could contribute toward other observed differences in our data. The analyses of Experiment 2 data showed that there was a significant difference in detection rates for 2000 vs. 1000 ms jumps in the review condition. However, the detection rates were similar for these magnitudes in the skip-ahead condition – see Figure 5, Figure 6d, and Figure 7. It is possible that these differences are a result of the involvement of memory. Re-view jumps as long as 1000 and 2000 ms are likely to convey an action/event that is different from the current ongoing event – thus resulting in an event boundary (Radvansky & Zacks, 2017; Zacks, 2013; Zacks & Swallow, 2007; Zacks & Tversky, 2001). Event boundaries have been shown to influence the organization of episodic memory and in the facilitation of temporal discrimination judgments in memory. Items farther in time in the temporal organization of memory are shown to be better differentiated compared to the items closer in the temporal organization (Eichenbaum, 2017; Pu, Kong, Ranganath, & Melloni, 2022; Yntema & Trask, 1963). Therefore, the detection rates could be better for the changes caused by 1000 and 2000 ms re-view jumps by virtue of the temporal organization of memory. This could explain why the detection rates were significantly different for the 1000 vs. 2000 ms in the re-view condition, but not the skip-ahead condition.

Moreover, the results of the optic flow similarity analyses also indicate that detection rates varied differently for skip-ahead versus re-view conditions. Specifically, the detection rates decreased as the optic flow similarity increased in the skip-ahead condition where overlap of the exact same content would not be available, whereas the opposite was true for the re-view condition where the exact same content was repeated. It is possible that these differences are the result of optic flow interactions with memory. The exact nature of such a mechanism is unclear and would require further investigation. Together, these data suggest a possible role of memory for higher detection rates in the re-view jumps. However, it should be noted that a purely memory-based account would be agnostic toward the detection behavior in the skip-ahead condition. Specifically, it cannot explain why detection rates decreased with increased optic flow similarity in the skip-ahead condition, thus providing an incomplete picture of the underlying mechanisms of spatiotemporal jump detections in film viewing.

A complementary explanation in addition to the memory-based explanation concerns our visual system's ability to rely on the knowledge of the unfolding future states. Studies on representational momentum using naturalistic videos have demonstrated a forward bias in the direction of motion when participants were asked to remember their last seen frame in a video clip (Thornton & Hayes, 2004). In addition, actors’ intentions and goals have also been shown to influence participant responses in the Representation Momentum paradigm (Hudson, Nicholson, Ellis, & Bach, 2016) – suggesting that anticipatory event knowledge influences visual processing. Moreover, prior exposure to temporal sequences has been shown to facilitate prediction of future states suggesting that memory could also help in the anticipation of future states (Baker, Dexter, Hardwicke, Goldstone, & Kourtzi, 2014). These results suggest that our visual processing benefits from the prediction of future states. Accordingly, our data also suggest the possibility of future prediction in detecting spatiotemporal jumps. The decrease in detection rates as a function of optic flow similarity implies that during skip-ahead jumps, a match between the input and the expected stimulus is likely to make the jump less detectable – thus demonstrating that the visual system relies on knowledge of unfolding visual information during film viewing. Although a low-level visual feature, optic flow has often been used in film editing to convey the underlying narrative. Cinematography techniques, such as camera motion and zoom, often use optic flow information to convey actors’ movement, intention, and goals. Accordingly, changes in transitions of optic flow reflect changes in lighting intensity, movement of objects, or change in locations and characters in the scene among the other factors (Bordwell, 2002; Cutting, DeLong, & Nothelfer, 2010). Often, disturbances in optic flow have been linked to event boundaries (Swallow, Kemp, & Simsek, 2018; Tan, 2018). Therefore, it is possible that top-down knowledge, such as event schema and scene semantics, could also influence jump detections through optic flow. Future work could benefit from further investigation on this front.

In addition to prediction-based processing, it is possible that knowledge about future states could aid jump detections through backward inferencing processes – wherein the missed information during the disruption is inferred retroactively instead of actively predicted. Recent work on event comprehension has argued that predictions sometimes require sustained mental effort and therefore might not be the default mode of event processing (Hymel, Levin, & Baker, 2016; Papenmeier, Brockhoff, & Huff, 2019; Radvansky & Zacks, 2017). For example, recent work by Hymel and colleagues (Hymel et al., 2016) showed people videos of actors that either did or did not contain an out of order action (e.g. using a screwdriver before picking it up). They found that participants were often unable to detect mis-ordered actions, thus arguing that people do not consistently compare predictions for the future with the current input. Moreover, another recent paper by Papenmeier and colleagues (Papenmeier et al., 2019) argued that completion of missing information is caused by a rapid backward inference process. Participants in their study watched a player running toward a soccer ball in preparation of kicking it. The clip was followed either by a causal continuation clip (e.g. the ball flying) or a non-causal continuation clip (e.g. a clip of the crowd). They found that participants were more likely to falsely detect seeing contact with the ball if the shot was followed by the causal continuation shot thus suggesting that missing information was filled in by a backward inference process. Together, these results suggest that prediction-based processing might not be always the default mode of event comprehension, contrasting with event segmentation theory (Zacks, 2013; Zacks & Swallow, 2007; Zacks & Tversky, 2001).

It is therefore possible that the skip-ahead disruptions in the present study are less likely to be noticed because it is easier to retroactively map the information after the jump to the current event model compared to the re-view jumps. Our results are currently limited in terms of making a distinction between predictive versus backward inference processes. However, it should be noted that backward inference in Hymel et al., 2016 and Papenmeier et al., 2019 was investigated using an event comprehension paradigm as opposed to the disruption paradigm that our study used. Furthermore, the mis-ordered/missing information in both studies contained crucial information at the event level (e.g.: screwdriver being picked up and the ball flying) as opposed to our study – where the missing information was created by jumping the video forward/backward in time. Given these differences, it is unclear how backward inferences would manifest in detecting spatiotemporal disruptions. Future work could benefit from a detailed investigation on this front.

Overall, our results suggest that our visual system benefits from both memory and knowledge of the future state during spatiotemporal disruptions in continuous film viewing.

## Appendix A. Appendix: Supplementary data

**Table A1.** Estimates of the GLME model on the detection data as a function of the change direction, magnitude and saccade duration. Each estimate provides the odds in favor of change in detection accuracy at level 1 compared to the level 0 of the corresponding predictor variable. Also shown are the 95% confidence intervals (CIs) for the estimates and their corresponding *P* values for tests checking if the estimates are significantly different compared to the chance level (odds ratio 1).

	Fixed effects			Random effects, *SD*	
Predictors	Odds ratios	CI	*P* value	By trial	By subject
Intercept [re-view, 500]	3.46	[2.24–5.33]	<0.001	0.14	0.38
Direction (skip-ahead)	0.59	[0.59–0.69]	<0.001	–	–
Magnitude (1000 msec)	1.66	[1.40–1.96]	<0.001	–	–
Saccade duration	1.00	[0.99–1.00]	0.442	–	–
Direction (skip-ahead): magnitude (1000)	0.67	[0.48–0.94]	0.02	–	–

CI, confidence interval.

**Table A2.** Estimates of the GLME model on the detection data as a function of the change direction, magnitude and saccade duration.

	Fixed effects			Random effects, *SD*	
Predictors	Odds ratios	CI	*P* value	By trial	By subject
Intercept [re-view, 1000]	5.54	[3.02–10.17]	<0.001	0.14	0.48
Direction (skip-ahead)	0.26	[0.20–0.34]	<0.001	–	–
Magnitude (2000 msec)	1.61	[1.23–2.09]	<0.001	–	–
Saccade duration	1.00	[0.99–1.01]	0.656	–	–
Direction (skip-ahead): magnitude (2000)	0.41	[0.24–0.70]	0.001	–	–

CI, confidence interval.

**Table A3.** GLME estimates of detection data as a function of direction and optic flow similarity.

	Fixed effects			Random effects, *SD*	
Predictors	Odds ratios	CI	*P* value	By trial	By subject
Intercept [re-view 1000, similarity = 0]	7.42	[5.67–9.70]	<0.001	0.15	0.43
Direction (skip-ahead)	0.43	[0.35–0.51]	**<0.001**	–	–
Magnitude (2000)	1.28	[1.01–1.64]	**<0.001**	–	–
Magnitude (500)	0.66	[0.56–0.79]	**<0.001**	–	–
Optic flow similarity	3.37	[1.75–6.47]	**<0.001**	–	–
Direction (skip-ahead): similarity	0.18	[0.09–0.38]	**<0.001**	–	–
Magnitude (2000): similarity	0.74	[0.23–2.34]	0.603	–	–
Magnitude (500): similarity	0.86	[0.49–1.53]	0.612	–	–

CI, confidence interval.
